# Contribution of Inhibitor of Differentiation and Estrogenic Endocrine Disruptors to Neurocognitive Disorders

**DOI:** 10.3390/medsci6030061

**Published:** 2018-08-03

**Authors:** Andrea Avecilla, Mayur Doke, Jeremy Jovellanos, Vincent Avecilla

**Affiliations:** 1 Department of Clinical Psychology, University of Massachusetts Dartmouth, North Dartmouth, MA 02747, USA; aavecilla@umassd.edu; 2 Department of Environmental Health Sciences, Florida International University, Miami, FL 33199, USA; mdoke001@fiu.edu; 3 Division of Pediatric Infectious Diseases, Bronx-Lebanon Hospital Center, Bronx, NY 10457, USA; jjovella@bronxleb.org

**Keywords:** endocrine disruptor, environmental health sciences, gene-environment, inhibitor of differentiation, neurocognitive disorders

## Abstract

The devastating growth in the worldwide frequency of neurocognitive disorders and its allied difficulties, such as decline in memory, spatial competency, and ability to focus, poses a significant psychological public health problem. Inhibitor of differentiation (ID) proteins are members of a family of helix-loop-helix (HLH) transcription factors. ID proteins have been demonstrated to be involved in neurodevelopmental and depressive diseases and, thus, may influence neurocognitive deficiencies due to environmental exposure. Previously, it has been demonstrated that environmental factors, such as estrogenic endocrine disruptors (EEDs), have played an essential role in the influence of various neurocognitive disorders such as Alzheimer’s, dementia, and Parkinson’s disease. Based on this increasing number of reports, we consider the impact of these environmental pollutants on ID proteins. Better understanding of how these ID proteins by which EED exposure can affect neurocognitive disorders in populations will prospectively deliver valuable information in the impediment and regulation of these diseases linked with environmental factor exposure.

## 1. Introduction

Neurocognitive disorders, which were earlier identified as dementia, denote a range of disorders that affect the brain and cause worsening in one or more cognitive areas. Deterioration in cognition need not only be damage to cognitive abilities, but also visible by others and tested by cognitive assessment. An estimated 1.2 million adults in the United States are diagnosed annually with a chronic neurocognitive disorder and, due to the aging population, these figures will increase in the coming decades. Additionally, an estimated 13–16 million individuals 18 years old and older are afflicted with these types of disorders. This type of disorder frequently affects older people, however, it is not part of the standard aging development and has the capability to affect young people as well. Neurocognitive disorders can affect language, memory, attention, social cognition, learning, and perception [1,2]. Today, there are main and trivial neurocognitive disorders that are reliant on how severely the symptoms influence an individual’s capability to function self-sufficiently in daily activities, such as Alzheimer’s, Parkinson’s, Creutzfeldt-Jakob disease, Huntington’s disease, and Vascular Dementia. Factors inducing various forms of neurocognitive ailments can include endocrine and metabolic ailments, substance abuse, infections, trauma, and nutritional deficiencies [2]. Presently, there is a necessity to recognize how factors such as estrogenic endocrine disruptors contribute to neurocognitive disorder susceptibility.

Estrogen, which belongs to a class of hormones, has been shown to have various functions including the regulation of metabolism and endocrine growth and development [3]. Furthermore, estrogen has been previously demonstrated to affect neurocognitive outcomes [3,4,5,6,7,8]. Due to this, neurocognitive disorders may be susceptible to estrogenic endocrine disruptors or EEDs. Endocrine disruptors modify hormone function or production, including anthropogenic chemicals, heavy metals, and phytoestrogens. More particularly, EEDs consist of arsenic, DES (Diethylstilbestrol), phthalates, bisphenol A (BPA), and polychlorinated biphenyls (PCBs) [9,10,11,12,13]. Studies have reported links between EED exposure and neurocognitive disorders [14,15,16]. Based on findings that demonstrate that a family of proteins (Inhibitor of Differentiation (ID)) has been associated with neurocognitive disorders [16], we will also highlight how EED exposure from the environment may potentiate neurocognitive outcomes via ID proteins. This review is concentrated on connecting EEDs to ID interactions leading to altered outcomes in neurocognitive disorders. More study in these capacities may uncover innovative or additionally applicable beneficial modalities and deliver approaches for neurocognitive outcomes with EED exposure.

## 2. Inhibitor of Differentiation

### 2.1. Structure and Function

Inhibitor of Differentiation is a family of proteins that consist of four genes (*ID1*, *ID2*, *ID3*, and *ID4*). The members of the ID family share an extensive amino acid sequence homology within their HLH (helix-loop-helix) domain (69–78%) [17,18]. It has been reported that ID protein acts as a transcription regulator, which regulate the transcription in a dominant-negative manner by dimerizing with basic HLH transcription factors like HEB, E47, and E12 [19,20,21]. ID proteins are pleiotropic proteins involved in the modulation of biological processes, such as cell proliferation and differentiation, cell cycle control, senescence, apoptosis or angiogenesis, and metastasis [22,23]. In case of the central nervous system, *ID1* and *ID3* are greatly expressed in the premature stages of nervous tissue development, but their levels decline in later stages [24,25]. Although ID1 and ID3 expression cease as tissue matures, *ID2* and *ID4* expression remain constant throughout the nervous tissue development [26,27,28]. Therefore, ID proteins play a very important role in nervous tissue biology. Intriguingly, reactivation of ID proteins in adult tissues is held responsible for the involvement of various cancers [29,30]. Researchers have shown the reactive oxygen species (ROS) induced ID protein-mediated cell proliferation and dysregulation of tissue biology in vitro and in vivo conditions [31,32,33]. Similarly, in another study Das et al. [34] have shown the exposure of 17-β estradiol (E2) and estrogenic endocrine disruptors (EED) like polychlorinated biphenyl 153 (PCB153) to vascular endothelial cells (ECs) increase ROS. While *ID3* is a redox-sensitive gene, it acts as an important determinant of the ROS-induced proliferation of E2 and ECs exposed to PCB153 [34,35,36]. Neurodegenerative disorders like Alzheimer’s and Parkinson’s are the main causes of dementia, and their symptoms worsen slowly over the time. The causes of neurodegenerative diseases apart from genetic mutations are mainly environmental factors, head injuries, depression, and hypertension. Researchers have shown that these causes are strongly interconnected with elevated levels of ROS [31,37,38]. The increased levels of ROS affect human tissues at a molecular level over time. As age increases, longer exposure to ROS may result in increasing tissue injury and severe disease symptoms. Since ID proteins are shown to be redox sensitive, we predict EEDs exposure boosts ROS-induced ID proteins levels, which may be responsible for the onset of neurocognitive deficiencies.

### 2.2. Inhibitor of Differentiation Proteins and Neurocognitive Disorders

There has been accumulative evidence demonstrating the role of ID proteins in various neurological deficiencies and disorders. One essential pathological hallmark of Alzheimer’s disease (AD) is the buildup of senile plaques largely comprised of amyloid β-peptide (Aβ) in the patients’ brains. Hung et al. [39] investigated if Aβ may stimulate Sonic hedgehog (SHH) expression and its essential mechanisms. Aβ25–35 induced *ID1*, which has been exhibited to stabilize HIF-1α (Hypoxia-inducible factor 1-alpha). Further, Aβ25–35-mediated induction of HIF-1α and SHH was both suppressed by ID1 siRNA. Taken together, the pathway inhibitor cyclopamine SHH and its antibody reduced Aβ cytotoxicity. Based on these lines of evidence, results showed a signaling pathway of Aβ → *ID1* → *HIF-1* → *SHH* [39]. Kitajima et al. [40] investigated ID2 mRNA-expressing cells in the adult mouse brain. Results showed that ID2 mRNA is identified in more diverse brain areas, including the amygdaloidal complex, globus pallidus, substantia nigra pars reticulata, suprachiasmatic nucleus caudate putamen, and the frontal part of the subventricular zone. These suggest that *ID2* may have a function in cognitive and neural activity. Additionally, expression of ID3 (moderate or low) was demonstrated alongside high expression in some specific areas, such as the molecular layer of the dentate gyrus. ID4 mRNA was detected in regions such like the lateral amygdaloidal nucleus. Based on these lines of evidence, the ID2 pattern expression is distinctive from those of partnering ID proteins [40]. Donepezil is a common medication for AD. Acetylcholinesterase (AChE) has been demonstrated to play a role in osteoblast function, however, the mechanism of AChE on osteoclastogenesis nevertheless remains uncertain. Donepezil reduced receptor activator of nuclear factor-κ B ligand (RANKL) expression in bone marrow macrophages (BMMs), resulting in the up-regulation of ID2 and inhibition of osteoclast differentiation with down-regulation of c-Fos (c-fos proto-oncogene). These particular results show that inhibition of osteoclast function due to donepezil prevents bone loss, which may suggest the chance that donepezil reduces fracture risk in patients with AD [41].

Uncertainties in rhythm circadian-related developments are recurrently established in anxiety and depressed-driven patients. Several genes have been recognized as factors for the progression of mood disorders. It was demonstrated that mild stress-stimulated chronic anhedonic behavior is connected amongst distressed diurnal alternation of the expression of *CLOCK*, *CRY2*, *PER3*, *PER1*, *REV-ERBα*, *ID2*, *ROR-β*, and *ROR-γ* in the mouse basolateral amygdala [42]. Furthermore, premature life abandonment increases risk for the psychopathological development through both childhood and adulthood, including anxiety disorders and depression. It was recently reported that epigenetic changes in DNA resulted in three genes predicting depression in maltreated children: *GRIN1*, *ID3*, and *TPPP*. Behavioral tests demonstrated that *GRIN1*, *ID3*, and *TPPP* gene expression were established to significantly predict behavioral alterations. These lines of evidence support the role of these genes in the etiology of anxiety and depressive phenotypes succeeding premature life stress [43]. Transcriptome wide association studies have, furthermore, been used to predict various epigenetic markers of depression. Methylation in four genes developed as predictors of depression: *ID3*, *NMDA*, *GRIN1*, and *TPPP* [44].

Similarly, Becker et al. [45] targeted a strategy to uncover foundations of variability in subcortical brain areas. Results demonstrated important improvement of genomic loci that affect the area of the hippocampus, a result that strongly passed the adjusted threshold for testing of multiple brain phenotypes. Investigation of individual single nucleotide polymorphisms (SNPs) also revealed a connection upstream of the *ID2* gene with rs7588305. Results show that targeting recognized regulatory regions indicates ways to comprehend the biology that links genotypes to phenotypes. [45]. Previously, Kepa et al. [46]. identified a network of HLH transcriptional regulators controlled by myelin transcription factor 1-like (MYT1L), as specified in the human brain and neural stem cells. It was demonstrated that *MYT1L* is essential for neuronal differentiation and identified *ID1* as a target. Furthermore, MYT1L prohibited *ID1* expression and induced expression of a large quantity of terminal differentiation genes. Consistent expression of *MYT1L* corresponded with neuronal maturation and linked *ID1* and *ID3* during the lifecycle. Additionally, genetic polymorphisms that abridged expression of MYT1L in the hippocampus caused enlarged *ID1* and *ID3* expression, reduced TCF4 (transcription factor 4) and NEUROD6 (neurogenic differentiation 6) levels and reduced gene expression involved in cancer, neurodegeneration, long-term potentiation, and synaptic transmission. As a result, these outcomes indicate that MYT1L controls memory-related developments by regulating a neuronal proliferation and differentiation mechanism of ID family proteins [46]. Additionally, six genes displaying at least differential expression among hemispheres (*BAIAP2*, *DAPPER1*, *LMO4*, *NEUROD6*, *ATP2B3*, and *ID2*) in a case-control association study in an initial Spanish sample of ADHD (Attention Deficit Hyperactivity Disorder) patients and control subjects. Outcomes support the contribution of genomic factors in the stability of ADHD in some of the populations examined and may supply influencing deviant cerebral lateralization in this condition [47].

Fetal Alcohol Spectrum Disorders, or FASD, signify a variety of antagonistic developmental ailments triggered by prenatal ethanol exposure (PrEE) from parental intake of alcohol. A mouse model of FASD demonstrated stable phenotypes transmitting by the male germline to the unexposed third generation. Global DNA methylation levels, modifications in ectopic intraneocortical connectivity, and up-regulation of neocortical *Rzrβ* and *ID2* was seen. These phenotypes may contribute to sensorimotor, cerebral, and communicative insufficiencies seen in individuals with FASD. Therefore, understanding the conceivable epigenetic mechanisms may uncover innovative targets for beneficial mediation of FASD [48].

### 2.3. Inhibitor of Differentiation Proteins and Estrogenic Endocrine Disruptors

Previously, we determined how *ID3* impacts obesity and metabolic health in response to environmental influences. Furthermore, we emphasized the understanding of how *ID3* may contribute to multidimensional diseases via metabolic perturbations [16,20]. PCBs are classified as a class of organochlorine mixtures that are tenacious in the environment and have the potential ability to disrupt the homeostasis of thyroid hormones (THs). In this study, Dong et al. [49] examined gene expression patterns (ID proteins including: ID1, ID2, and ID3) in juvenile Japanese flounder (*Paralichthys olivaceus*) following exposure to environmentally-applicable concentrations of a commercial PCB mixture, Aroclor 1254 (Monsanto Corporation, Monsanto Lot KI-6024, San Luis, MO, United States). Exposure to Aroclor 1254 increased follicular cell height, colloid depletion, and hyperplasia. Changes in mRNA expression levels of IDs were identified in the liver and kidney, which may be connected with a reduction in plasma THs levels. ID2 mRNA expression in the liver demonstrated an increase constructed via dose-dependence, signifying that this may function as a constant marker for thyroid-disrupting chemical (TDC) exposure. Overall, results suggest that applicable levels of Aroclor 1254 produce noteworthy thyroid disruption [49].

Micro-vascular lesions from endothelial cell dysfunction are formed in various areas of patients with complex chronic diseases such as the lung, brain, retina, and kidney. The mechanisms dependable for starting micro-vascular injury remain weakly distinct, while factors have been suggested, including oxidative stress induced by environmental chemicals. Heightened neovascularization has been associated in the progression or development of proliferative vascular lesions. Previously, support for how ROS via PCBs may contribute to neo-vascular phenotype development with the aim of revealing the function of environmental chemicals in endothelial dysfunction with a concentration on *ID3* has been shown. Results demonstrated that PCB-induced ROS intermediated neo-vascular phenotype additionally depended on *ID3* and Pyk2 (Protein-tyrosine kinase 2). Furthermore, treatment of PCB153 enlarged the dimension of endothelial spheroids with circumstances that function on behalf of stem cell spheroid clonal selection. Elevated ID3 protein expression compared with a larger amount of oxidative DNA damage marker 8-OHdG in blood vessels. The results provide the potential role of *ID3* in regulating development of micro-vascular lesions and vascular endothelial cell survival prompted by environmental chemicals such as PCB153 [34,35]. Furthermore, another study determined if in utero exposure to BPA stimulated reproductive tract irregularities in the adult male testis. Adult C57/BI6 males mice testis histopathology, anogenital distance, and sex-organ weights were exposed in utero through oral gavage to sesame oil, 50 µg/Kg BPA, 1000 µg/kg BPA, or 2 µg/kg diethylstilbestrol (DES) as a positive control from gestational days 10 to 16 and examined. Adult mRNA levels of genes connected with differentiation and sexual maturation, *GATA4* and *ID2*, were lower only in testes exposed to DES. At the molecular level, DES exposure via in utero, not BPA, leads to reduced mRNA expression of genes linked with Sertoli cell differentiation [50].

## 3. Influence of Estrogenic Endocrine Disruptors Exposure on Neurocognitive Disorders

Exposure to estrogenic endocrine disruptors has been previously shown in various population and animal studies. Previously Bell et al. [51] tested the effects of PCBs on prenatal or juvenile individuals. The effects had differential results on age-dependent and sex behaviors. Females demonstrated different social and anxiety behavior in adolescence, while males exhibited small but significant changes in socio-sexual preferences in adulthood [51]. Exposure to low levels of PCBs is known to lead to anxious behavior in offspring mice, both young and adult. At further advanced life stages, an effect on the mouse brain of neuronal stress induced by the AB peptide was evaluated. Significant impairment in long-duration memory was identified in the mice treated lacatational with non-dioxin-like PCBs (NDL-PCBs). Early exposure to low levels of NDL-PCBs stimulates late neuronal susceptibility to amyloid tension [52]. Exposure to PCBs may result in changed procreative behaviors in adulthood. Rat dams (pregnant) were injected on gestational days with PCB mixture Aroclor 1221 at one of two doses. Females were unaffected, but males treated with A1221 presented decreased indicators of anxiety [53]. Researchers have also studied subchronic embryonic exposure to PCBs concerning anxiety-associated components. Exposure induced behavioral deficiencies at seven days post-fertilization was detected. Outcomes demonstrated that exposed larvae had enhanced edge preference relative to the control. Furthermore, larvae that were exposed reacted contrarily to a graphical risk comparative to control larvae [54]. Comprehensive gene expression profiles in cerebellar exhibiting the highest suggestive stimulation of anxiety-like behavior has been warranted via male mice. Outcomes demonstrated alterations in the expression of genes in the neurons of the PCB-exposed mice [55]. Furthermore, studies have analyzed the relationship between work-related PCB exposures. Analysis including individuals with their plasma PCBs collected via bio-monitoring and the psychological syndromes assessed with a consistent screening method. Results showed greater PCB burdened individuals had greater depression but not anxiety syndrome [56].

REST (Repressor element 1-silencing transcription factor) plays a significant function in neuronal phenotype development in neural progenitor cells and nonneuronal cells. Chronic exposure to the PCB mixture Aroclor-1254 triggered cell death. PCB mixture reduced acetylation of the histone proteins H3 and H4. These outcomes collectively indicate that A-1254 services its toxic influence via REST by down-regulating synapsin-1 and weakening H3 and H4 acetylation [57]. Changes in calcium signaling, thyroid hormones, and neurotransmitters have been postulated as potential mechanisms for neurotoxicity development in animal models through PCBs. Gene expression levels in the hippocampus and cerebellum from PNDs (postnatal days) animals were determined in the study. In the cerebellum, transcripts demonstrated modification at PND7 compared to transcripts at PND14 by Aroclor-1254 exposure, with only one transcript disturbed at both ages. Aroclor-1254-induced genomic changes were greater in the hippocampus than the cerebellum. The outcomes suggest that neurotoxic effects via PCB stimulated disruption of standard ontogenetic design of nervous system growth and development by regulating signaling pathways [58].

Individuals were exposed to PCBs and polychlorinated dibenzofurans (PCDFs) due to absorption of polluted cooking oil in Taiwan. Neurocognitive performance in individuals exposed to PCDFs and PCBs with that of unexposed was compared. Evaluation of neurocognitive examinations was directed. In exposed men, outcomes were comparable to the reference group; conversely, exposed women had diminished functioning in attention and digit span visual memory span, and verbal memory recalls. The study demonstrated neurocognitive deficits in certain dose-dependent aspects of attention, learning ability, and visual memory in women previously exposed to PCBs and PCDFs however, not in exposed men [59]. Samples taken from women and blood samples during birth, pregnancy, from the umbilical cord, and breast milk were tested for PCB congeners and organochlorine pesticides. PCB153 levels in these media were comparatively low in relation to other studies. Measurements of PCBs in samples taken during the second trimester of pregnancy, at birth, and in the umbilical cord were strongly associated. Particular measurements of PCB153 and PCB180 among those subjects with concluded sampling of blood samples from mothers and cord samples were significantly correlated. Maternal blood measurement can reliably estimate the fetal exposure to PCBs during the second trimester. This assessment is consistent for PCB 153 and PCB 180 [60].

Additionally, BPA was examined in urine samples from women in the first and third trimesters of pregnancy. Psychomotor alongside cognitive growth was measured using psychologist-based scales. BPA exposure concentrations in the highest tectile were associated with a reduction of psychomotor scores at one year of age, however no associations with psychomotor outcomes at four years were shown. Exposure to BPA was linked with a higher risk of ADHD symptoms at four years old. Overall, the results indicate that BPA exposure prenatally does not disturb cognitive growth up to four years old. Links are demonstrated with ADHD-correlated signs and psychomotor development at initial ages, but do not appear to remain until later ages [61]. Furthermore, hormone-induced alterations in brain composition and role indicate that EED exposure may be connected with sex-specific modifications in behavior. BPA has shown to alter androgen, estrogen, and thyroid hormone signaling pathways. Mothers with measurable prenatal urinary BPA were correlated with relatively higher affecting and expressing depressed behavior, somatic problems, and ODD (Oppositional Defiant Disorder) behaviors in boys. The results indicate higher behavior problems in school aged boys but not girls [62]. Additionally, it has been shown that communicative effects of developmental exposure to a low dose of BPA with detail to the maternal environment, time of exposure, and sex and age. During both testing ages, females whom were exposed presented suggestion of higher anxiety and were less disposed to search a new environment. The results specify that sexually dimorphic behaviors are delicate to endocrine disruption during important developmental periods, specifically during important early neonatal stages [63]. BPA exposure during gestation has been additionally suggested as a risk element of neurobehavioral disorders. Exposure to a regular low-dose of BPA during pregnancy tested offspring of mice. Mice who were exposed had offspring that had increased anxiety-like behavior [64].

## 4. Interaction of Inhibitor of Differentiation Proteins and Estrogenic Endocrine Disruptors in Neurocognitive Disorders

**Figure 1:**
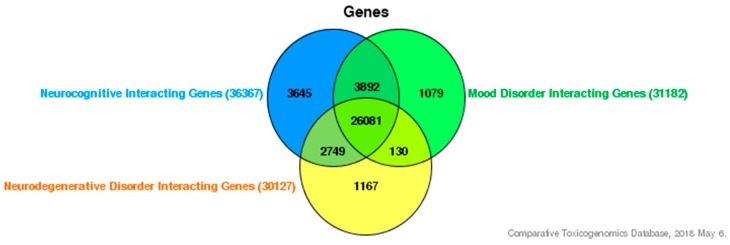
Interacting genes of neurocognitive categories. Shown are 26,081 genes that interact within the three categories.

**Figure 2:**
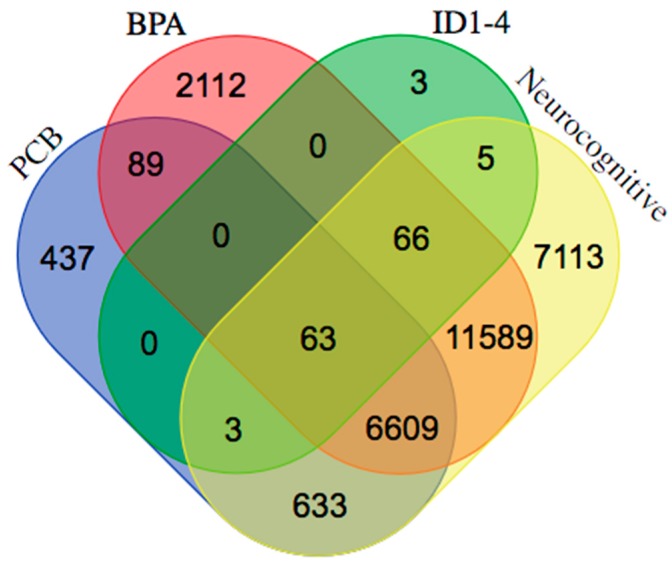
Venn diagram displaying interacting genes between estrogenic endocrine disruptors (EED) (polychlorinated biphenyls (PCBs) and bisphenol A (BPA)), ID1-4, and neurocognitive disorders. Results show 63 overlapping genes [65,71].

**Figure 3:**
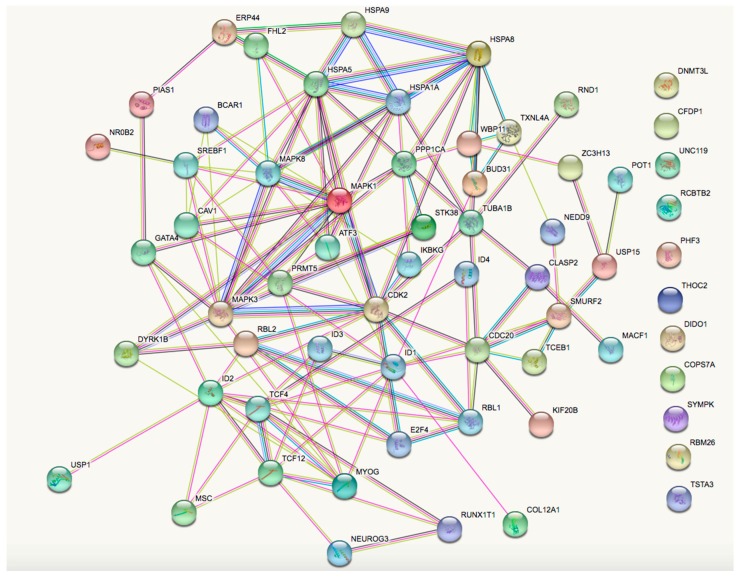
Gene network of 63 common interacting genes between EEDs, ID proteins, and neurocognitive disorders [66].

In order to investigate how ID proteins and environmental exposures affect neurocognitive deficiencies at a genetic level, we used various publicly accessible databases to help support our understanding. First, we used the Comparative Toxicogenomic Database (CTD) [65], a research tool that determines chemical-gene and chemical-disease association, to help decipher gene-environment or gene-EED interactions involved in the production of various neurocognitive deficiencies. To demonstrate an overlapping interaction between neurocognitive disorders, we established a list of neurocognitive-interacting genes (36,367), mood disorder interacting-genes (31,182), and neurodegenerative-interacting genes (30,127). As shown in Figure 1, 26,081 genes are commonly interacting. We also selected interacting genes with estrogenic endocrine disruptors PCB and BPA. We established a gene list for both: PCBs having 7825 interacting genes and BPA having 20,504 interacting genes. Since ID proteins are our candidate gene group, we also generated a list of overlapping genes between each of the ID proteins (ID1, ID2, ID3, and ID4) and established interacting genes between the two chosen EEDs and neurocognitive disorder categories. We established that 63 genes interact with EEDs, our ID proteins, and neurocognitive disorders, as shown in Figure 2 and Table 1 [65]. To demonstrate an interaction between these 63 genes; we furthermore inputted them in STRING [66], a recognized database that can provide protein-to-protein interactions. Overall, the interactions contain either indirect (functional) or direct (physical) connections, which stem from interactions gathered from various databases, computational prediction, and from the transfer of knowledge between organisms. As seen in Figure 3, STRING provides a network of these 63 common genes and additionally provides connections between all the genes [66,67]. Furthermore, to demonstrate the importance of these genes we also used the Kyoto Encyclopedia of Genes and Genomes pathway, a collection of pathways representing knowledge for molecular reaction, interaction, and relation networks. We established that these genes were involved in 71 biological/molecular pathways [68]. The top five are represented in Table 2 and it is revealed that these pathways offer functionality in neurocognitive disorders [69,70,71,72,73,74].

## 5. Conclusions

Inhibitor of Differentiation proteins have been demonstrated to be involved with neurocognitive disorders. Studies have reported links between neurocognitive disorders and exposure to EEDs, such as PCBs and BPA. Based on the evidence discussed in this review, we have demonstrated that exposure to EEDs may activate ID proteins to alter molecular mechanisms, additionally changing neurocognitive disorder outcomes. As an emerging topic with restricted information, it is important to address the limitations of this study by conducting further studies to assess how EED exposure and ID proteins play a role in neurocognitive perturbations from both a biological and pathological perspective. With further epidemiological, in vivo, and in vitro research, the establishment of molecular mechanisms can be more defined and understood. This essential information will allow research scientists, toxicologists, and public health professionals to discover novel avenues for the prevention and treatment of these types of disorders.

## Figures and Tables

**Table 1:** Gene symbols and gene names of 63 overlapping EED-Inhibitor of Differentiation (ID) protein-neurocognitive genes.

Gene Symbol	Gene Name
*ATF3*	Activating transcription factor 3 (ATF3)
*BCAR1*	Breast cancer anti-estrogen resistance protein 1, Cas family scaffolding protein (BCAR1)
*BUD31*	BUD31 homolog (BUD31)
*CAV1*	Caveolin 1 (CAV1)
*CDC20*	Cell division cycle 20 (CDC20)
*CDK2*	Cyclin-dependent kinase 2 (CDK2)
*CFDP1*	Craniofacial development protein 1 (CFDP1)
*CLASP2*	Cytoplasmic linker associated protein 2 (CLASP2)
*COL12A1*	Collagen type XII α 1 chain (COL12A1)
*COPS7A*	Constitutive photomorphogenesis 9 signalosome subunit 7A (COPS7A)
*DIDO1*	Death inducer-obliterator 1 (DIDO1)
*DNMT3L*	DNA methyltransferase 3 like (DNMT3L)
*DYRK1B*	Dual-specificity tyrosine phosphorylation regulated kinase 1B (DYRK1B)
*E2F4*	E2F transcription factor 4 (E2F4)
*ELOC*	Elongin c (ELOC)
*ERP44*	Endoplasmic reticulum protein 44 (ERP44)
*FHL2*	Four and a half LIM domains 2 (FHL2)
*GATA4*	Global transcription factor binding protein 4 (GATA4)
*HSPA1A*	Heat shock protein family A member 1A (HSPA1A)
*HSPA5*	Heat shock protein family A member 5 (HSPA5)
*HSPA8*	Heat shock protein family A member 8 (HSPA8)
*HSPA9*	Heat shock protein family A member 9 (HSPA9)
*ID1*	Inhibitor of DNA binding 1, helix-loop-helix (HLH) protein (ID1)
*ID2*	Inhibitor of DNA binding 2, HLH protein (ID2)
*ID3*	Inhibitor of DNA binding 3, HLH protein (ID3)
*ID4*	Inhibitor of DNA binding 4, HLH protein (ID4)
*IKBKG*	Inhibitor of kappa light polypeptide gene enhancer in B-cells, kinase gamma (IKBKG)
*KIF20B*	Kinesin family member 20B (KIF20B)
*MACF1*	Microtubule-actin crosslinking factor 1 (MACF1)
*MAPK1*	Mitogen-activated protein kinase 1 (MAPK1)
*MAPK3*	Mitogen-activated protein kinase 3 (MAPK3)
*MAPK8*	Mitogen-activated protein kinase 8 (MAPK8)
*MSC*	Musculin (MSC)
*MYOG*	Myogenin (MYOG)
*NEDD9*	Neural precursor cell expressed, developmentally down-regulated 9 (NEDD9)
*NEUROG3*	Neurogenin 3 (NEUROG3)
*NR0B2*	Nuclear receptor subfamily 0 group B member 2 (NR0B2)
*PHF3*	PHD finger protein 3 (PHF3)
*PIAS1*	Protein inhibitor of activated STAT (signal transducer and activator of transcription) 1 (PIAS1)
*POT1*	Protection of telomeres 1 (POT1)
*PPP1CA*	Protein phosphatase 1 catalytic subunit α (PPP1CA)
*PRMT5*	Protein arginine methyltransferase 5 (PRMT5)
*RBL1*	RB (retinoblastoma protein) transcriptional corepressor like 1 (RBL1)
*RBL2*	RB transcriptional corepressor like 2 (RBL2)
*RBM26*	RNA binding motif protein 26 (RBM26)
*RCBTB2*	RCC1 (regulator of chromosome condensation 1) and BTB domain containing protein 2 (RCBTB2)
*RND1*	RNA binding motif protein 26 family GTPase 1 (RND1)
*RUNX1T1*	Runt related transcription factor 1 (RUNX1T1)
*SMURF2*	SMAD specific E3 ubiquitin protein ligase 2 (SMURF2)
*SREBF1*	Sterol regulatory element binding transcription factor 1 (SREBF1)
*STK38*	Serine/threonine kinase 38 (STK38)
*SYMPK*	Symplekin (SYMPK)
*TCF12*	Transcription factor 12 (TCF12)
*TCF4*	Transcription factor 4 (TCF4)
*THOC2*	THO complex 2(THOC2)
*TSTA3*	Tissue-specific transplantation antigen P35B (TSTA3)
*TUBA1B*	Tubulin α 1b (TUBA1B)
*TXNL4A*	Thioredoxin like 4A (TXNL4A)
*UNC119*	unc-119 Lipid Bind Chaperone (UNC119)
*USP1*	Ubiquitin specific peptidase 1 (USP1)
*USP15*	Ubiquitin specific peptidase 15 (USP15)
*WBP11*	WW domain binding protein 11 (WBP11)
*ZC3H13*	Zinc finger CCCH-type containing 13 (ZC3H13)

**Table 2:** Top five pathways with 63 common interacting genes with EEDs, ID, and neurocognitive disorders.

Pathway Name	Gene Count	Matching Genes in Network (Nodes)
TGF-β signaling pathway	9	*E2F4, ID1, ID2, ID3, ID4, MAPK1, MAPK3, RBL1, SMURF2*
Toxoplasmosis	6	*HSPA1A, HSPA8, IKBKG, MAPK1, MAPK3, MAPK8*
Focal adhesion	6	*HSPA1A, HSPA5, MAPK1, MAPK3*
Viral carcinogenesis	6	*FHL2, IKBKG, MAPK1, MAPK3, MAPK8*
MAPK (Mitogen-activated protein kinase) signaling pathway	6	*BCAR1, CAV1, MAPK1, MAPK3, MAPK8, PPP1CA*
